# Advanced Optimization of Surface Characteristics and Material Removal Rate for Biocompatible Ti6Al4V Using WEDM Process with BBD and NSGA II

**DOI:** 10.3390/ma16144915

**Published:** 2023-07-09

**Authors:** Anbazhagan Nagadeepan, Govindarajalu Jayaprakash, Vagheesan Senthilkumar

**Affiliations:** 1 Department of Mechanical Engineering, SRM TRP Engineering College, Trichy 621105, Tamilnadu, India; trpvsk12@gmail.com; 2 Department of Mechanical Engineering, Saranathan College of Engineering, Trichy 620012, Tamilnadu, India; jayaprakashcad@gmail.com

**Keywords:** WEDM, Box–Behnken design, NSGA II, material removal rate, surface roughness, titanium alloy

## Abstract

Machining titanium alloy (Ti6Al4V) used in orthopedic implants via conventional metal cutting processes is challenging due to excessive cutting forces, low surface integrity, and tool wear. To overcome these difficulties and ensure high-quality products, various industries employ wire electrical discharge machining (WEDM) for precise machining of intricate shapes in titanium alloy. The objective is to make WEDM machining parameters as efficient as possible for machining the biocompatible alloy Ti6Al4Vusing Box–Behnken design (BBD) and nondominated sorting genetic algorithm II (NSGA II). A quadratic mathematical model is created to represent the productivity and the quality factor (MRR and surface roughness) in terms of varying input parameters, such as pulse active (T_on_) time, pulse inactive (T_off_) time, peak amplitude (A) current, and applied servo (V) voltage. The established regression models and related prediction plots provide a reliable approach for predicting how the process variables affect the two responses, namely, MRR and SR. The effects of four process variables on both the responses were examined, and the findings revealed that the pulse duration and voltage have a major influence on the rate at which material is removed (MRR), whereas the pulse duration influences quality (SR). The tradeoff between MRR and SR, when significant process factors are included, emphasizes the need for a reliable multi-objective optimization method. The intelligent metaheuristic optimization method named nondominated sorting genetic algorithm II (NSGA II) was utilized to provide pareto optimum solutions in order to achieve high material removal rate (MRR) and low surface roughness (SR).

## 1. Introduction

Ti6Al4V or Titanium Grade 5 is biocompatible, which means that it is well tolerated by the human body, making it suitable for medical implants such as orthopedic implants [1], dental implants [2], and prosthetics [3]. The superior corrosion resistance, formability, and weldability lead to the extensive use of titanium alloys in a wide range of industries such as the aerospace [4], automotive [5,6], chemical [7], and marine industries [8]. Titanium and its alloys are difficult to machine/process and expensive when utilized in conventional machining methods because of their high chemical reactivity (causing tool deformation) [9,10] and poor thermal conductivity [11,12]. In several industries, wire electric discharge machining (WEDM), which utilizes thermo-mechanical energy, can process with excellent accuracy [13] and provide a good surface finish [14,15]; thus, it is often employed to deal with such high-strength materials. In the WEDM process, a conductive wire (electrode) travels continually, and the heat results in an electric spark that erodes the conductive work material [16,17]. During the process, dielectric fluid is utilized as a medium for ionization to create the electrical arc across the workpiece and the wire (electrode). The steady flow of dielectric fluid acts as a cooling agent and helps in flushing the removed material away. The heat energy from these electrical sparks removes material in the form of micro-debris.

The WEDM equipment’s conservative technical data or the operator’s expertise was traditionally used to choose the appropriate parameters for the maximum material removal rate and minimum surface roughness during the EDM process, which resulted in inconsistent machining performance. Devarasiddappa et al. [18] focused on reducing the surface roughness of Ti6Al4V alloy processed using WEDM. They obtained a 2.6% reduction in SR for ideal process variables of the WEDM process. Muhammad Umar Farooq et al. [19] manufactured convex and concave shapes in Ti6Al4V using the WEDM technique and found that servo voltage, wire feed, pulse-on time, and pulse-off time affect the precision of geometrical profiles and the diameter of corners. The pulse duration was shown to be the most important variables driving overcut in convex profiles. Chaudhari et al. [20] employed Taguchi’s L9 array to machine Ti6Al4V alloy using the WEDM technique and found that pulse duration and current have an impact on MRR and SR. Lin et al. [21] emphasized the benefits of using the gray Taguchi technique to optimize a number of quality variables such as electrode depletion, MRR, and overcut for micro-EDM finish quality of the Ti6Al4V alloy. According to the experimental findings, the peak current and pulse duration play a key role in the micro-EDM of Ti6Al4V alloy. Priyadarshini et al. [22] studied the use of Ti6Al4V alloy using EDM for various pulse parameters and chose MRR, SR, and TWR (tool wear rate) as output factors utilizing a Taguchi orthogonal array for the experiment.

Material removal rate and surface roughness were the output parameters utilized by Gupta et al. [23] while using the WEDM process to machine a Ti6Al4V alloy, whereas the process parameters were pulse duration, wire speed, servo voltage, wire tension, pulse current, and feed rate. They observed that pulse duration has a significant effect on MRR and SR. When using the WEDM method to cut Ti6Al4V alloy, Mouralova et al. [24] evaluated the current, voltage, pulse duration, and wire speed as the process parameters. They determined using experiments that a quicker pulse-on time and longer pulse-off time resulted in a better surface quality. Ti6Al4V was studied as a work material by Pramanik et al. [25] and machined utilizing the WEDM method while taking the effect of wire tension, pulse-on time, and pulse-off time as process parameters into consideration. They reached the conclusion that shorter pulse-on times and longer pulse-off times produced better results. Bisaria and Shandilya [26] studied the influence of pulse duration and spark gap voltage on MRR (material removal rate) and SR (surface roughness). They discovered that, although MRR and SR values decreased with a rise in voltage and pulse-off time, they increased drastically with a rise in pulse-on time. Many experimental investigations have sought to investigate the impact of various process factors on the performance of the WEDM machining. The majority of the experiments, however, had limitations on the variety of process variables they were able to investigate or the range of values they could investigate, and it can be suggested that there is still scope for improvement in terms of collecting data by piloting an in-depth and comprehensive investigation of the impact of a wide variety of WEDM process variables and their levels on the productivity and surface quality generated when processing Ti6Al4V alloy.

The appropriate process parameters is to be selected carefully for sustainable production, and the parameters associated with WEDM for the machining of the Ti6Al4V alloy have been optimized by a number of researchers [27,28]. To collect data from experiments with the minimum waste of time, money, raw materials, etc., systematic experiment design is crucial. Among the tools for designing experiments are Taguchi techniques employing an orthogonal array, response surface methodology (RSM), and fractional factorial design. A comprehensive review of the literature revealed that several researchers from all over the world have contributed to the process parameter optimization of the WEDM method for the machining of titanium-based alloys. WEDM is a process with multiple inputs and multiple outputs that has complex relationships on each parameter and how they interact with each other. When multiple objectives are dealt with, numerous conflicting situations arise that require a resolution. The best representation of this tradeoff is optimum pareto points obtained through evolutionary optimization which finds the solution near the global optimum with less time and computing effort. Thus, there arises a need for multi-response optimization [29], including gray relational analysis (GRA) [30,31], heat transfer search (HTS) algorithm [32,33], teacher learning-based algorithm [34], particle swarm algorithm [35], genetic algorithm [36], and artificial neural networks [37], which have been tested in order to find the tradeoff solution in terms of optimized process parameters.

Deb et al. [38] implemented NSGA II (non-sorted genetic algorithm II), a newly developed approach that was designed to handle multi-objective problems and search for a variety of solutions that efficiently reflect the tradeoff between contrasting objectives. NSGA II can effectively explore the solution space, maintain diversity, and provide optimum or near-optimal solutions for the optimization of two conflicting objectives, namely, maximum material removal rate (MRR) and minimal surface roughness in WEDM. AISI 5160 steel [39], high-speed steel [40], and AISI D3 tool steel [41,42] are a few materials that are processed in WEDM whose results are optimized using NSGA II. In the literature, limited studies can be found on minimizing SR and maximizing MRR in WEDM of Ti6Al4V alloy using the NSGA II algorithm, which can select the most dominant solution. 

The goal of this study was to optimize the machining settings in order to maximize MRR while minimizing SR. Accordingly, this study can be broken into three sections. The first section involves conducting experiments based on Box–Behnken design and collecting data regarding the responses of MRR and SR to various input parameters, including pulse active (T_on_) time, pulse inactive (T_off_) time, peak amplitude of current (A), and applied servo voltage (V). In the second section, the effect and influence of the parameters on the responses are examined and discussed. The third section develops the multi-objective metaheuristic NSGA II algorithm, which can search for the most optimal parameter for the conflicting objective, i.e., maximizing MRR and minimizing SR. Comparisons are made between the results obtained using these different methods, and validation is carried out via the use of confirmation tests.

## 2. Materials and Methods

**Figure 1:**
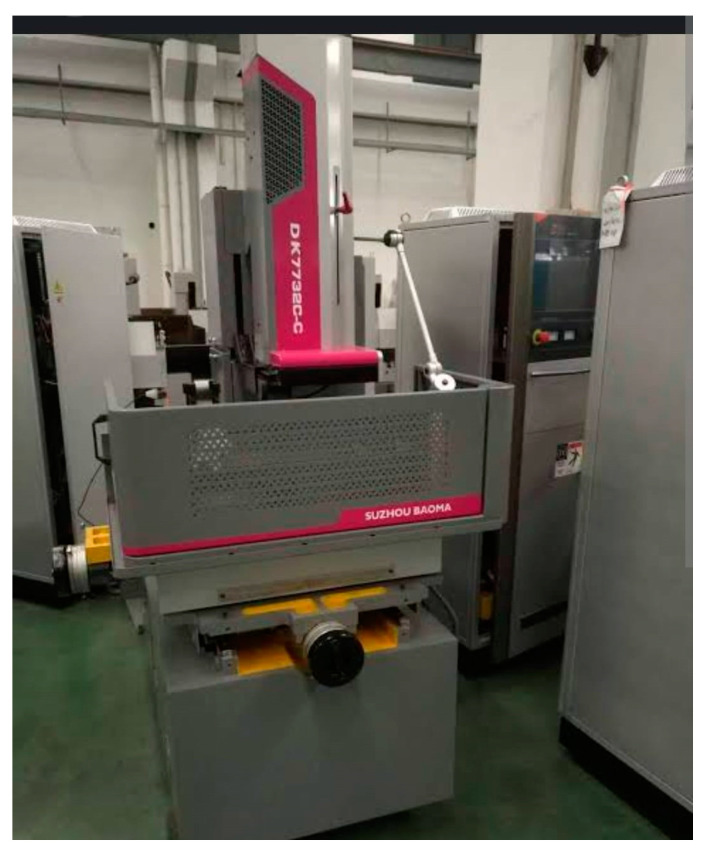
DK7732 wire-cut electro-discharge machine.

**Figure 2:**
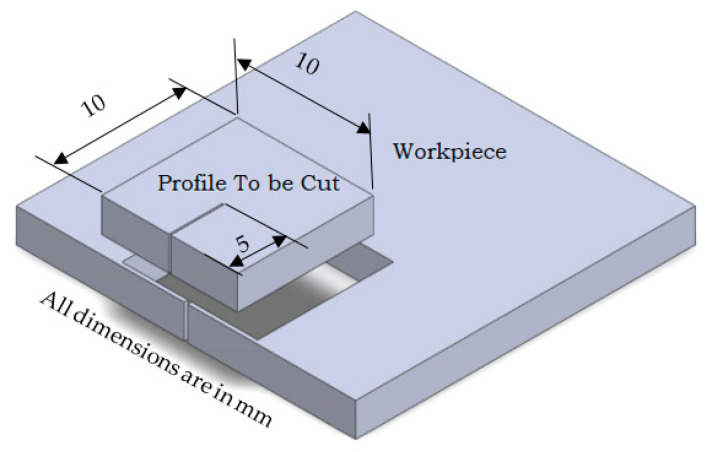
Profile.

In this work, a CNC wire-cut EDM DK7732C-C manufactured by Suzhou Baoma Numerical Control Equipment Co., Ltd. (Suzhou and China) with 3 kVA power and a BMW 3000 controller (Figure 1) was used to cut the profile shown in Figure 2 on a commercial titanium Ti6Al4V alloy of 5 mm thickness. Table 1 presents a list of the chemical components of the titanium Ti6Al4V alloy utilized in this investigation. Deionized water was utilized to eject the waste particles produced during the machining process, and a Ф 0.25 mm zinc-coated brass wire was utilized as the electrode for cutting purposes.

RSM is an abbreviation for response surface methodology, which is a powerful mathematical and statistical tool that can investigate and optimize complicated systems. Researchers have extensively utilized the Box–Behnken design (BBD), which is a form of response surface methodology (RSM), in order to optimize experimental trials [43]. The Box–Behnken design is advantageous since it does not contain any points at the extremities of the cubic region produced by the two-level factorial combinations that are either costly to test or difficult to test due to physical restrictions in experimentation [44]. It is widely used in a variety of sectors, including agrochemicals, pharmaceuticals, and bioprocessing [45]. 

The experiments used a two-level design with four parameters, namely, pulse active time (T_on_) ranging from 110 to 120 µs, pulse inactive time (T_off_) ranging from 50 to 60 µs, applied servo voltage (V) ranging from 40 to 50 V, and peak amplitude of current (A) ranging from 40 to 42 A. The Box–Behnken design was employed to generate a total of 29 experiments, and the corresponding values of the response characteristics were determined. Table 2 depicts the range of input parameters that were chosen on the basis of preliminary pilot experiments and an extensive investigation of the literature.

The response characteristics evaluated in the present study were the MRR and average SR. The arithmetic average roughness, i.e., the average deviation of the surface profile from the mean line within the evaluation length was considered for measuring surface roughness, expressed in micrometers. An SJ-210-Mitutoyo surf test equipped with a 2 µm stylus tip diamond indenter was used to measure the SR at three different locations. The average of all three values was then selected. Equation (1) was used to determine the MRR after the machining time was measured using a stopwatch [46,47].

(1) $$MRR=\frac{L\;H}{t},$$

where L is the cutting length is constant (45 mm), H is the height of the workpiece (5 mm), and t is the machining time (recorded using stopwatch in min). 

## 3. Results and Discussion

### 3.1. Response Surface Methodology (RSM)

A statistical and mathematical approach called response surface methodology (RSM) can be used to optimize and investigate the interaction between a number of input variables (factors) and one or more output variables (responses). It is commonly used in the field of experimental design and process optimization, which is particularly useful when the association between the input variables and the response variables is intricate and nonlinear. By systematically varying the levels of the input variables and measuring the corresponding response, RSM helps to model and understand the relationship and, subsequently, determine the optimal values of the input variables to achieve desirable responses.

#### 3.1.1. Multivariate Analysis (ANOVA)

Table 3 depicts the BBD and experimental findings of the machining capabilities characterizing the WEDM process. For a 5% significance level (α = 0.05), the “*p*-value” must be less than or equal to 0.05 to be considered significant, and the backward elimination method excludes irrelevant factors. The model’s potential for predicting an optimal response value for correlation coefficient R was evaluated in the most favorable conditions possible. Regression analysis was used to determine the optimum region to investigate the responses under study by fitting mathematical models to the experimental data. The data from the experiment runs were analyzed and obtained with the use of Design expert software V 13.0, and the significance of each coefficient for MRR and SR is depicted in Table 4 and Table 5.

An experimental relationship expressed by a second-order polynomial equation with interaction terms was utilized to fit the data from the BBD model. The final result of coded factors is shown in (Equations (2) and (3)).

(2) $$\begin{array}{ll} MRR=4.60+1.02 & \times \;A+0.3522\times B-0.1804\times C-0.0177\times D+0.1490\times AB-0.0721\times AC-0.3641\times AD\\ & -\;0.0467\times BC+0.0152\times BD+0.1394\times CD+0.4589\times A^{2}-0.0627{\;\times \;B}^{2}-0.0200{\;\times \;C}^{2}+0.1211\times D^{2}. \end{array}$$

(3) $$\begin{array}{ll} Ra=+3.64+0.445 & \times \;A+0.2419\times B-0.0718\times C-0.0447\times D+0.0625\times AB-0.1099\times AC-0.1031\times AD\\ & +\;0.0994\times AC-0.0558\times BD+0.1027\times CD+0.2176\times A^{2}-0.0321{\;\times \;B}^{2}-0.0796{\;\times \;C}^{2}\\ & +\;0.0346\times D^{2}. \end{array}$$

Analysis of variance (ANOVA) was implemented to analyze the data, and Table 4 and Table 5 show the significance of the experimental findings for different models along with the related *p*-values. Four linear correlation coefficients (A, B, C, and D), six interaction coefficients (AB, AC, AD, BC, BD, and CD), and four quadratic coefficients (A^2^, B^2^, C^2^, and D^2^) were found to be substantial. The *p*-values of each model term were investigated to depict the interaction patterns between the variables; for *p* < 0.0001, the model’s F-value of 18.5 for MRR and 10.02 for SR showed that it was very significant. Due to the relative pure error, the lack of fit F-value of 0.8983 for MRR and 0.5147 for SR was not significant (*p* = 0.0702). Overall, unless the model is adequately fit to validate its suitability for use, inadequate or inaccurate results may be obtained through the investigation and optimization of the fitted response surface [48,49]. The *p*-value is used to determine the significance of each coefficient and the degree of interaction between variables. Effects characterized by *p* < 0.05 are considered significant. The degree of correlation between the values observed and predicted is better when the significance level is greater [50]. The “Pred R-squared” of 0.9175 and the “Adj R-squared” of 0.9342 for MRR and the “Pred R-squared” of 0.9021 and the “Adj R-squared” of 0.9278 for SR were reasonably compatible in the current examination. In addition, the correlation coefficients with a high value (R^2^ for MRR of 0.9687 and for SR of 0.9542) showed a significant relationship between the values of the anticipated and experimental responses. The low CV value (5.42% for MRR and 4.52% for SR) made it evident that there were minor deviations between the predicted and experimental results, and it also demonstrated that the experiments were very precise and reliable. The adequate precision ratio in the current investigation was determined to be 14.91 for MRR and 12.13 for SR, indicating a sufficient indicator, and the present model may be utilized to move about the design space.

#### 3.1.2. Diagnostic Graphs

**Figure 3:**
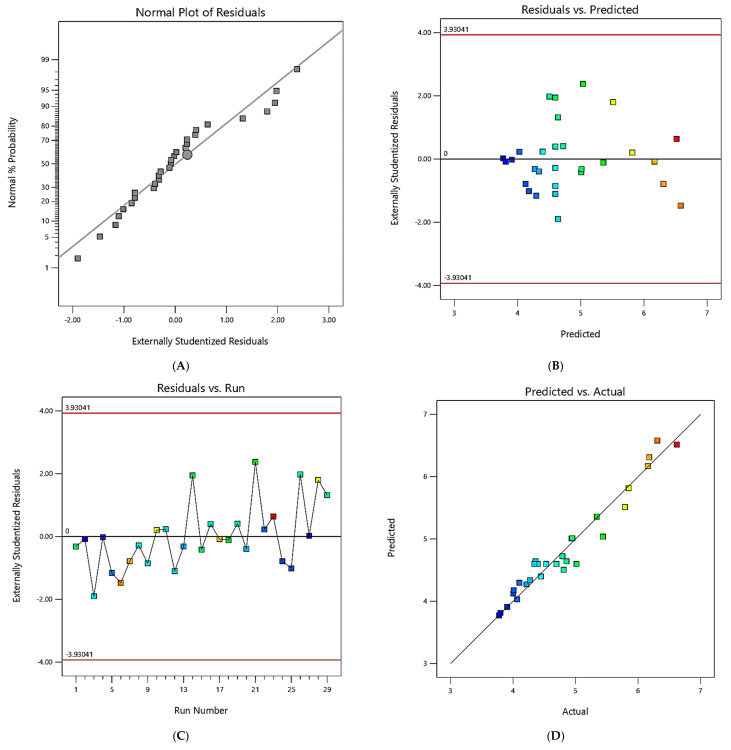

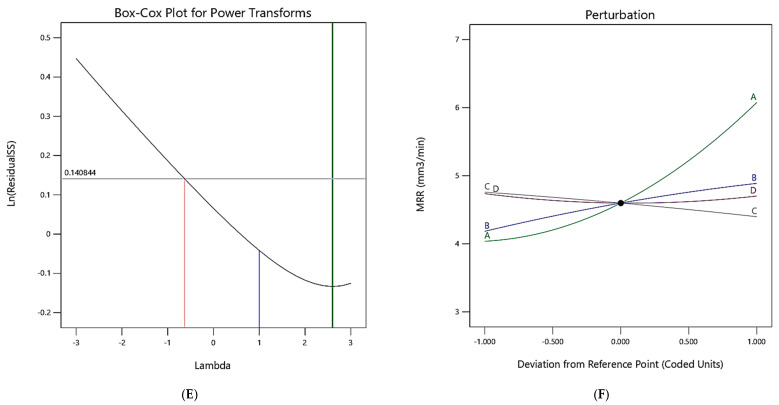
Diagnostic graphs for the Box–Behnken model for MRR: (**A**) Normal plot of residuals; (**B**) residuals (versus) predicted; (**C**) Residuals (versus) run; (**D**) Predicted (versus) actual; (**E**) Box–Cox plot—power transformations; (**F**) Perturbation. Letters A, B, C and D are the process (variable) parameters. The colored squares represent the residual of each run.

**Figure 4:**
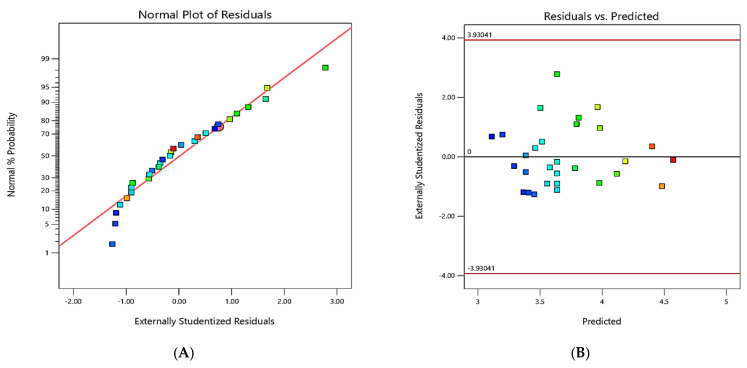

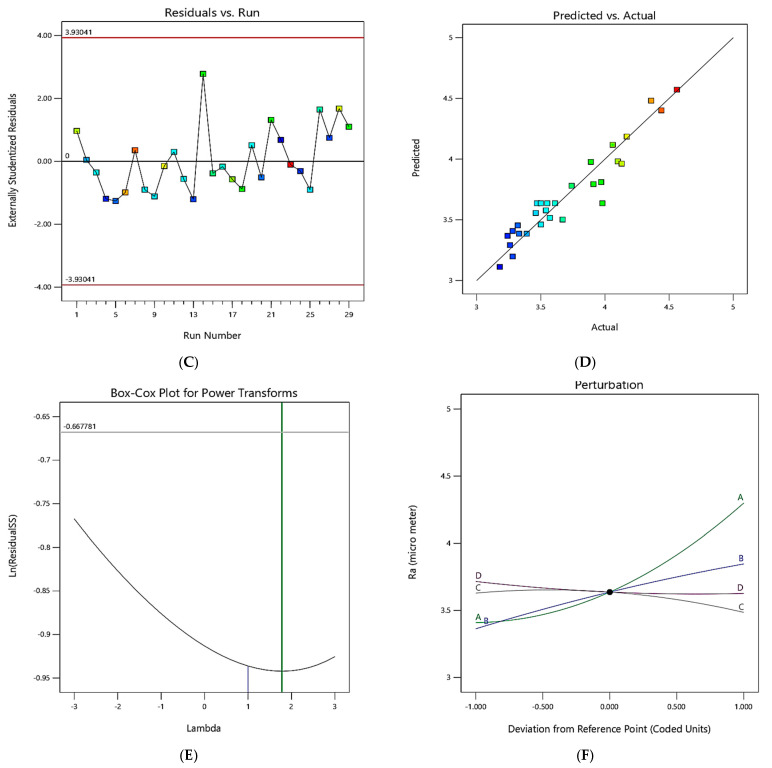
Diagnostic graphs for the Box–Behnken model for SR: (**A**) normal plot of residuals; (**B**) residuals (versus) predicted; (**C**) residuals (versus) run; (**D**) predicted (versus) actual; (**E**) Box–Cox plot—power transformations; (**F**) perturbation. (Letters A, B, C and D are the process (variable) parameters. The colored squares represent the residual of each run).

The data statistics are shown in Figure 3 and Figure 4; analyzing the experimental and the model observation diagnostic plots enabled the determination of the models’ accuracy. Figure 3A and Figure 4A show the residual plots for the responses, which exhibited a normal% probability distribution, whereby the points were logically closer to the straight line, with no significant deviations; thus, the current model significantly improved the interaction between the response and the process variables.

The predicted values were extremely similar to the observed values, as shown in Figure 3B and Figure 4B. By establishing a satisfactory fit of the model, the externally studentized residuals vs. experimental runs were examined, and the results showed that all of the data points fit inside the limits (Figure 3C and Figure 4C). For each run, the values of the predicted and actual responses were regularly distributed with almost straight lines, as shown in Figure 3D and Figure 4D. Figure 3E,F and Figure 4E,F show the Box–Cox plots (for power transformations of variables), indicating that the perturbation of variables was within the specified range.

#### 3.1.3. 3D Response Surface Plot

In order to understand the major impacts of the two variables, as well as their interactions, it is useful to compare the effects of the two components using 3D surface plots.

**Figure 5:**
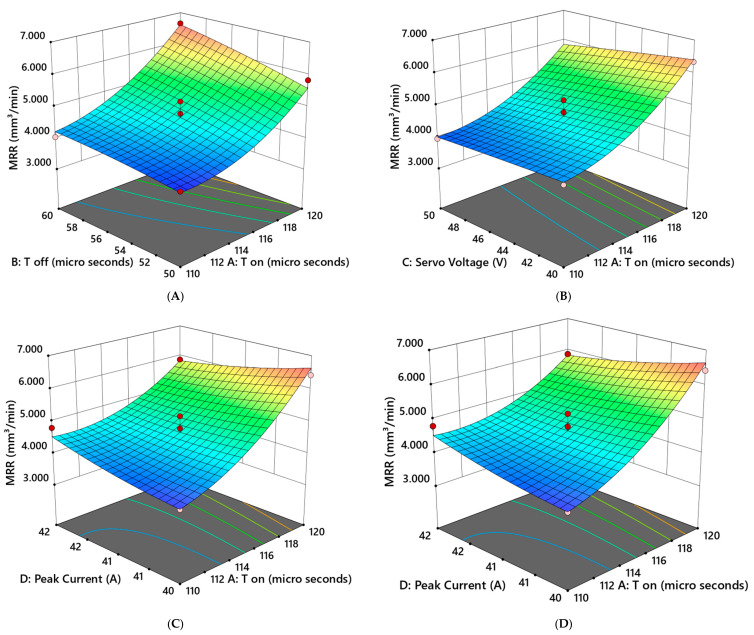

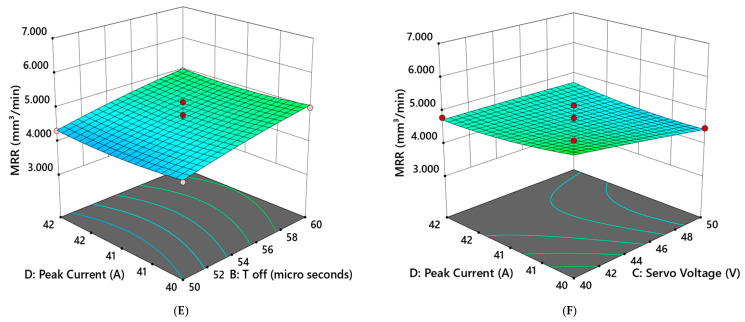
The 3D response surface plot for MRR: (**A**) MRR vs. T_on_ and T_off_; (**B**) MRR vs. servo voltage and T_on_; (**C**) MRR vs. peak current and T_on_; (**D**) MRR vs. servo voltage and T_off_; (**E**) MRR vs. peak current and T_off_; (**F**) MRR vs. peak current and servo voltage. (Where Red color representing maximum value, Green color represents intermediate values and blue color representing minimum values).

Figure 5 illustrates the 3D response surface plot for the MRR, from which it can be observed that the maximum MRR (6.62 mm^3^/min) was recorded for maximum T_on_ (120 µs) and T_off_ (60 µs) but medium voltage (45 V) and current (41 A). When the titanium (Ti6Al4V) alloy was processed using WEDM, the combined effects of T_on_ with T_off_, T_on_ with current, and T_on_ with servo voltage (Figure 5A–C) resulted in better spark creation and improved characteristics, which increased workpiece vaporization and melting, thereby resulting in increased MRR. Therefore, the combination of higher values for these input parameters resulted in an increase in the EDM process’s overall productivity. In the case of the interactions of T_off_ with current and T_off_ with voltage (Figure 5D,E), it is evident that there was little variation in these two factors, along with a gradual increase in MRR. The interaction of current and voltage (Figure 5F) had a negligible impact on the WEDM productivity.

**Figure 6:**
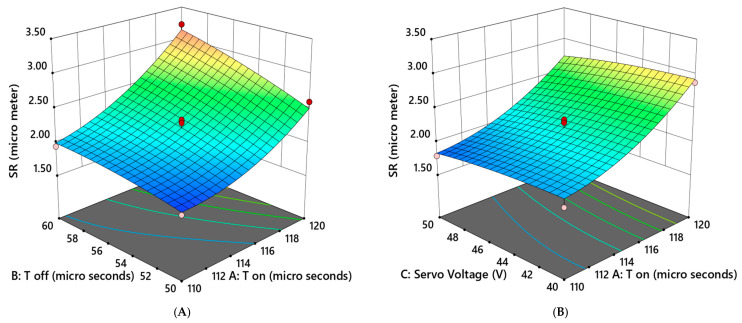

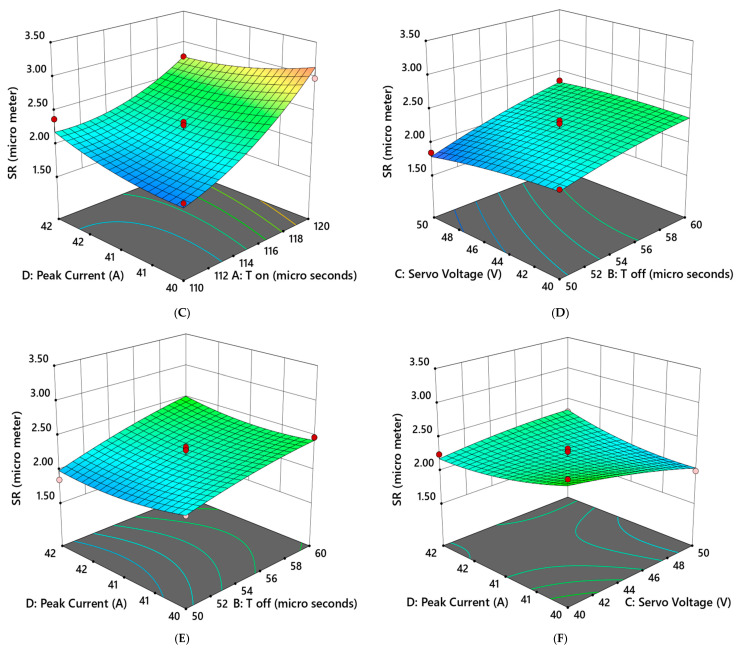
The 3D Response surface plot for SR: (**A**) SR vs. T_on_ and T_off_; (**B**) SR vs. servo voltage and T_on_; (**C**) SR vs. peak current and T_on_; (**D**) SR vs. servo voltage and T_off_; (**E**) SR vs. peak current and T_off_; (**F**) SR vs. peak current and servo voltage. (Where Red color representing maximum value, Green color represents intermediate values and blue color representing minimum values).

From the 3D response surface plot of SR (Figure 6), it can be observed that the surface roughness was minimum (3.18 µm) for maximum voltage (50 V), minimum T_off_ (50 µs), and medium T_on_ (115 µs) and current (41 A). In general, it can be observed that interactions among the various machining parameters were much simpler than the simple rule of thumb, and their combined impact on the process response was quite ambiguous. Figure 6A–C show how roughness varied with T_on_ and T_off_, T_on_ and current, and T_on_ and servo voltage. According to the figure, superior quality (i.e., smaller SR values) could essentially be achieved by combining lower values of T_on_ and T_off_, T_on_ and current, and T_on_ and servo voltage in contrast to their impact on process productivity (MRR). In the case of the interaction of T_off_ with current and T_off_ with voltage (Figure 6D,E), it is evident that there was little variation in these two factors, along with a gradual decrease in SR for decreasing inputs. The interaction of current and voltage (Figure 6F) had a negligible impact on the roughness.

### 3.2. Multi-Objective Optimization Using Nondominated Sorting Generic Algorithm II (NSGA II)

NSGA II is a powerful multi-objective optimization algorithm that uses nondominated sorting to find the optimal solutions that satisfy multiple objectives simultaneously. In this case, the objectives were to maximize MRR and minimize SR for WEDM parameters, which are dependent on T_on_, T_off_, V, and A. However, MRR and SR are conflicting objectives, and optimizing one may lead to the degradation of the other. To address this issue, NSGA II practices elitist nondominated selection to obtain a nondominated set of solutions. The nondominated solutions are selected on the basis of the ranking of the nondominated level, which is performed using the crowd comparing operator and crowd distance. These methods help to maintain population diversity and prevent the algorithm from premature convergence to a local optimum.

The NSGA II procedure is outlined below.

Step 1: On the basis of the maximum and minimum values of the input parameters, initialize the population (N);
Step 2: Determine the fitness functions for every individual, including MRR and surface roughness;
Step 3: Sort the initialized population using nondominated criteria;
Step 4: Choose the individuals on the basis of crowding distance and ranking, and then produce offspring via crossover operations with a factor of 0.95 and mutation operations with a factor of 0.01;
Step 5: Add the populations of the parents and offspring together, and determine who belongs in the next generation according to ranking and crowding distance;
Step 6: If the maximum generation (500) has been achieved, stop; if not, return to Step 4.

Due to the nature of MRR and SR conflicts and their dependence on T_on_, T_off_, PC, and SV, this can be resolved by assigning a negative sign (indicating minimization) to MRR.

Thus, maximize MRR = minimize (−MRR) = f(T_on_, T_off_, SV, PC) and minimize SR = f(T_on_, T_off_, V, A), subject to 110 ≤ T_on_ ≤ 120 µs, 50 ≤ T_off_ ≤ 60 µs, 40 ≤ SV ≤ 50 V, and 40 ≤ PC ≤ 42 A.

**Figure 7:**
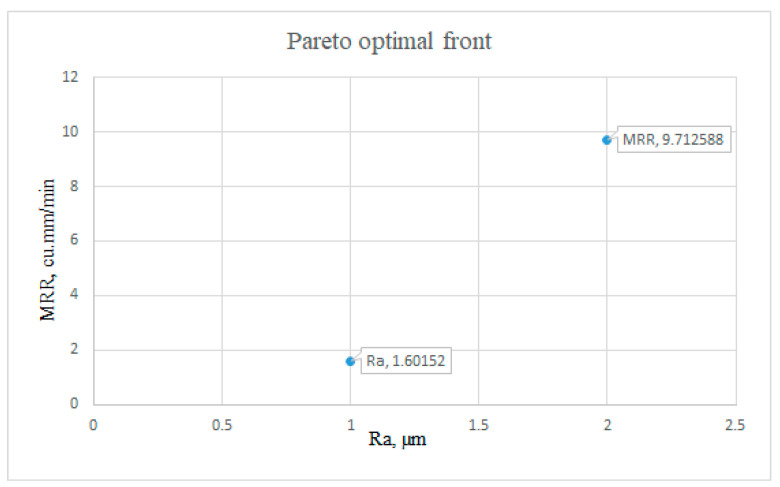
Pareto optimal front.

ANOVA was performed using Equations (2) and (3) to obtain objective functions for MRR and SR. Table 4 represents the pareto optimal solutions obtained in a study aimed at achieving superior surface finish and increased material removal rate (MRR). The study was conducted by varying four machining parameters, namely, pulse active (T_on_) time, pulse inactive (T_off_) time, servo voltage, and peak amplitude of current, using a pareto fraction of 0.1 and a population size of 1000. Table 6 includes 23 solution sets, with the optimal solution being solution number 2. Each solution set was characterized by corresponding values of T_on_, T_off_, servo voltage, peak current, MRR (in mm^3^/min), and SR (surface roughness in μm). MRR and SR were the two objective functions obtained after conducting ANOVA. The table shows that the values of T_on_, T_off_, servo voltage, and peak current varied across the solution sets, leading to different values of MRR and SR. Figure 7 illustrates the 23 results of the pareto optimal front, highlighting a better convergence of the results. Depending on the specific product requirements, any solution from the set in Table 4 can be acceptable. The most desirable set, on the other hand, was chosen with the primary goal of achieving a superior productivity factor and quality factor (MRR and roughness); in this case, solution 2 was preferable to other pareto optimal solution sets.

## 4. Optimization Validation

The final step was to validate and confirm the improvement in the performance characteristic using the optimum level of the process variables obtained using NSGA II. A confirmation experiment was performed on optimal solution set 2, and the results of the verification experiment using the optimum procedure variables are shown in Table 7, along with a comparison of NSGA II for the optimal and experimental process variables. According to Table 7, the MRR dropped by 1.06%, while the SR increased by 1.54%.

## 5. Conclusions

The current study utilized BBD/NSGA II to optimize MRR and SR in the WEDM of Ti6Al4V alloy. Mathematical equations, which were quadratic in nature, were obtained as a function of WEDM parameters for MRR and SR. On the basis of the results of the study, the following conclusions can be drawn:

- This study provides valuable insights into the optimization of the WEDM process for biocompatible titanium alloy Ti6Al4V.
- The nonlinear behavior in the responses of MRR and SR in the WEDM of Ti6Al4V alloy was found to be suitable for modeling, characterized by a quadratic model.
- The models developed for MRR and SR were adequate with a high F-value and reasonably decent association with the trial results (R^2^ of 96.87% for MRR and 95.42% for SR).
- The pulse active (T_on_) time was identified as the most influential WEDM parameter with respect to MRR and SR, achieving a maximum percentage, followed by pulse inactive time (T_off_).
- The NSGA II optimization technique proved to be a more effective method for optimizing multiple objectives, and the NSGA II technique provided pareto optimal solutions that offered most favorable balance between surface characteristics and material removal rate.
- The best process variables obtained using the optimization technique NSGA II were a T_on_ of 120 µs, T_off_ of 57 µs, servo voltage of 50 V, and peak current of 40 A.

## Figures and Tables

**Table 1:** Chemical constituents of Ti6Al4V alloy.

Element	Ti	Al	V	Fe	O
Weight (%)	~90	~6	~4	Max 0.25	Max 0.2

**Table 2:** Control factors and their levels.

Parameters and Their Levels	−1 Level	+1 Level
Pulse active (T_on_) time (µs)	110	120
Pulse inactive (T_off_) time (µs)	50	60
Servo voltage (SV) (V)	40	50
Peak amplitude of current (PC) (A)	40	42

**Table 3:** Box–Behnken design and response values.

Expt No.	Input Parameters				Responses	
	A—T_on_ (µs)	B—T_off_ (µs)	C—SV (V)	D—PC (A)	MRR (mm^3^/min)	SR (µm)
1.	115	60	45	40	5.011	2.48
2.	110	55	45	40	3.849	1.92
3.	115	55	50	42	4.41	2.30
4.	110	55	50	41	3.957	1.79
5.	115	50	40	41	4.103	2.09
6.	120	55	45	40	6.423	2.98
7.	120	55	40	41	6.345	2.88
8.	115	55	45	41	4.51	2.29
9.	115	55	45	41	4.423	2.13
10.	120	55	45	42	5.873	2.77
11.	115	55	50	40	4.484	2.00
12.	115	55	45	41	4.429	2.04
13.	115	50	45	42	4.327	1.85
14.	115	55	45	41	5.173	2.34
15.	115	60	45	42	4.956	2.42
16.	115	55	45	41	4.792	2.31
17.	120	55	50	40	6.145	2.88
18.	115	60	40	40	5.446	2.57
19.	115	55	40	42	4.805	2.25
20.	115	50	45	40	4.36	2.11
21.	115	55	40	40	5.512	2.60
22.	115	50	50	41	4.209	1.85
23.	120	60	45	41	6.639	3.23
24.	110	55	40	41	4.087	1.84
25.	110	60	45	41	4.041	1.94
26.	110	55	45	42	4.808	2.38
27.	110	50	45	41	3.916	1.75
28.	120	50	45	41	5.838	2.60
29.	115	60	50	41	5.006	2.35

**Table 4:** ANOVA results for MRR.

Source	Sum of Squares	Df	Mean Square	F-Value	*p*-Value
Model	17.75	14	1.27	18.5	<0.0001
A—T_on_	11.88	1	11.88	173.37	<0.0001
B—T_off_	1.42	1	1.42	20.68	0.0005
C—SV	0.3557	1	0.3557	5.19	0.0589
D—PC	0.004	1	0.004	0.0588	0.8119
AB	0.0888	1	0.0888	1.3	0.274
AC	0.0181	1	0.0181	0.264	0.6154
AD	0.5798	1	0.5798	8.46	0.0114
BC	0.0076	1	0.0076	0.1108	0.7441
BD	0.001	1	0.001	0.0148	0.905
CD	0.0911	1	0.0911	1.33	0.2681
A^2^	1.34	1	1.34	19.61	0.0006
B^2^	0.0251	1	0.0251	0.3664	0.5546
C^2^	0.0026	1	0.0026	0.0379	0.8484
D^2^	0.0929	1	0.0929	1.36	0.2636
Residual	0.9591	14	0.0685		
Lack of fit	0.6636	10	0.0664	0.8983	0.5972
Pure error	0.2955	4	0.0739		
Cor total	18.71	28			

**Table 5:** ANOVA results for SR.

Source	Sum of Squares	Df	Mean Square	F-Value	*p*-Value
Model	3.93	14	0.2806	10.02	<0.0001
A—T_on_	2.26	1	2.26	80.8	<0.0001
B—T_off_	0.6684	1	0.6684	23.86	0.0002
C—SV	0.0564	1	0.0564	2.01	0.1777
D—PC	0.0256	1	0.0256	0.9138	0.3553
AB	0.0156	1	0.0156	0.5579	0.4675
AC	0.042	1	0.042	1.5	0.2409
AD	0.0465	1	0.0465	1.66	0.2183
BC	0.0344	1	0.0344	1.23	0.2866
BD	0.0136	1	0.0136	0.4866	0.4969
CD	0.0494	1	0.0494	1.76	0.2054
A^2^	0.3023	1	0.3023	10.79	0.0054
B^2^	0.0066	1	0.0066	0.2349	0.6354
C^2^	0.041	1	0.041	1.46	0.2464
D^2^	0.0076	1	0.0076	0.2706	0.6111
Residual	0.3921	14	0.028		
Lack of fit	0.2206	10	0.0221	0.5147	0.8203
Pure error	0.1715	4	0.0429		
Cor total	4.32	28			

**Table 6:** Pareto optimum frontal solutions.

S. No.	T_on_	T_off_	Servo Voltage	Peak Current	MRR	SR
1.	120	59	50	40	8.995111	1.789038
2.	120	57	50	40	9.712588	1.60152
3.	120	57	50	40	9.630805	1.608884
4.	120	60	50	40	8.612424	1.987299
5.	120	60	50	40	8.573463	1.99937
6.	120	58	50	40	9.160236	1.714015
7.	120	57	50	40	9.553325	1.61439
8.	120	58	50	40	9.085653	1.746697
9.	120	57	50	40	9.643118	1.60284
10.	120	59	50	40	9.037575	1.77747
11.	120	60	50	40	8.686113	1.954955
12.	120	60	50	40	8.463633	2.066013
13.	120	59	50	40	8.835108	1.866152
14.	120	60	50	40	8.488892	2.045831
15.	120	58	50	40	9.323258	1.665676
16.	120	59	50	40	8.7235	1.911485
17.	120	57	50	40	9.479039	1.623467
18.	120	59	50	40	8.791831	1.87836
19.	120	59	50	40	8.694218	1.928456
20.	120	59	50	40	9.040872	1.76309
21.	120	60	50	40	8.508329	2.03119
22.	120	58	50	40	9.403723	1.647672
23.	120	60	50	40	8.43774	2.074575

**Table 7:** The results of the NSGA verification experiment.

Input Parameters				Responses		
T_on_	T_off_	Servo Voltage	Peak Current		MRR	SR
120	57	50	40	NSGA-II	9.712	1.60
				Experiment	9.609	1.62
				% Deviation	1.06%	1.54%

## Data Availability

The study did not report any data.

## References

[1] Gogolewski D., Kozior T., Zmarzły P., Mathia T.G. (2021). Morphology of Models Manufactured by SLM Technology and the Ti6Al4V Titanium Alloy Designed for Medical Applications. Materials.

[2] Meto A., Conserva E., Liccardi F., Colombari B., Consolo U., Blasi E. (2019). Differential efficacy of two dental implant decontamination techniques in reducing microbial biofilm and re-growth onto titanium disks in vitro. Appl. Sci..

[3] Jhong Y.T., Chao C.Y., Hung W.C., Du J.K. (2020). Effects of Various Polishing Techniques on the Surface Characteristics of the Ti-6Al-4V Alloy and on Bacterial Adhesion. Coatings.

[4] Li S., Zhang D., Liu C., Shao Z., Ren L. (2021). Influence of dynamic angles and cutting strain on chip morphology and cutting forces during titanium alloy Ti-6Al-4 V vibration-assisted drilling. J. Mater. Process. Technol..

[5] Lui E.W., Medvedev A.E., Edwards D., Qian M., Leary M., Brandt M. (2021). Microstructure modification of additive manufactured Ti-6Al-4V plates for improved ballistic performance properties. J. Mater. Process. Technol..

[6] Liu C., Liu D., Zhang X., Yu S., Zhao W. (2017). Effect of the ultrasonic surface rolling process on the fretting fatigue behavior of Ti-6Al-4V alloy. Materials.

[7] Gurrappa I. (2003). Characterization of titanium alloy Ti-6Al-4V for chemical, marine and industrial applications. Mater. Charact..

[8] Wang C.P., Wang H.Z., Ruan G.L., Wang S.H., Xiao Y.X., Jiang L.D. (2019). Applications and prospects of titanium and its alloys in seawater desalination industry. IOP Conf. Ser. Mater. Sci. Eng..

[9] Benea L., Simionescu-Bogatu N. (2021). Reactivity and Corrosion Behaviors of Ti6Al4V Alloy Implant Biomaterial under Metabolic Perturbation Conditions in Physiological Solutions. Materials.

[10] Swain S., Kumar R., Panigrahi I., Sahoo A.K., Panda A. (2022). Machinability performance investigation in CNC turning of Ti–6Al–4V alloy: Dry versus iron-aluminium oil coupled MQL machining comparison. Int. J. Lightweight Mater. Manuf..

[11] Pervaiz S., Deiab I., Rashid A., Nicolescu C.M. (2014). Experimental and Numerical Investigation of Ti6Al4V Alloy machinability using TiAlN Coated Tools. Trans. North Am. Manuf. Res. Inst. SME.

[12] García-Martínez E., Miguel V., Martínez-Martínez A., Manjabacas M.C., Coello J. (2019). Sustainable Lubrication Methods for the Machining of Titanium Alloys: An Overview. Materials.

[13] Karmiris-Obratański P., Papazoglou E.L., Leszczyńska-Madej B., Zagórski K., Markopoulos A.P. (2021). A Comprehensive Study on Processing Ti–6Al–4V ELI with High Power EDM. Materials.

[14] Kumar R., Roy S., Gunjan P., Sahoo A., Sarkar D.D., Das R.K. (2018). Analysis of MRR and Surface Roughness in Machining Ti-6Al-4V ELI Titanium Alloy Using EDM Process. Procedia Manuf..

[15] Hareesh K., Nalina Pramod K.V., Linu Husain N.K., Binoy K.B., Dipin Kumar R., Sreejith N.K. (2021). Influence of process parameters of wire EDM on surface finish of Ti6Al4V. Mater. Today Proc..

[16] Rathi P., Ghiya R., Shah H., Srivastava P., Patel S., Chaudhari R., Vora J. (2019). Multi-response optimization of Ni55. 8Ti shape memory alloy using taguchi–grey relational analysis approach. Proceedings of the Recent Advances in Mechanical Infrastructure: Proceedings of the ICRAM 2019.

[17] Chaudhari R., Ayesta I., Doshi M., Khanna S., Patel V.K., Vora J., De Lacalle L.N.L. (2022). Effect of Multi-walled carbon nanotubes on the performance evaluation of Nickel-based super-alloy–Udimet 720 machined using WEDM process. Int. J. Adv. Manuf. Technol..

[18] Devarasiddappa D., Chandrasekaran M., Arunachalam R. (2020). Experimental investigation and parametric optimization for minimizing surface roughness during WEDM of Ti6Al4V alloy using modified TLBO algorithm. J. Braz. Soc. Mech. Sci. Eng..

[19] Farooq M.U., Ali M.A., He Y., Khan A.M., Pruncu C.I., Kashif M., Ahmed N., Asif N. (2020). Curved profiles machining of Ti6Al4V alloy through WEDM: Investigations on geometrical errors. J. Mater. Res. Technol..

[20] Vora J., Prajapati N., Patel S., Sheth S., Patel A., Khanna S., Ayesta I., de Lacalle L.L., Chaudhari R. (2022). Multi-response optimization and effect of alumina mixed with dielectric fluid on WEDM process of Ti6Al4V. Recent Advances in Mechanical Infrastructure: Proceedings of the ICRAM 2021.

[21] Lin M., Tsao C., Huang H., Wu C., Hsu C. (2015). Use of the grey-Taguchi method to optimise the micro-electrical discharge machining (micro-EDM) of Ti-6Al-4V alloy. Int. J. Comput. Integr. Manuf..

[22] Priyadarshini M., Pal K. (2016). Multi-objective optimisation of EDM process using hybrid Taguchi-based methodologies for Ti-6Al-4V alloy. Int. J. Manuf. Res..

[23] Gupta N.K., Pandey p., Mehta S., Swati S., Mishra S.K., Tom K.J., Prasad A., Gupta S., Tyagi R. (2019). Characterization of ABS Material in Hybrid Composites: A Review. Advances in Engineering Design Lecture Notes in Mechanical Engineering.

[24] Mouralova K., Kovar J., Karpisek Z., Kousa P. (2016). Optimization Machining of Titanium Alloy Ti-6Al-4V by WEDM with Emphasis on the Quality of the Machined Surface. J. Manuf. Technol..

[25] Pramanik A., Basak A.K. (2019). Effect of wire electric discharge machining (EDM) parameters on fatigue life of Ti-6Al-4V alloy. Int. J. Fatigue.

[26] Bisaria H., Shandilya P. (2018). Experimental studies on electrical discharge wire cutting of Ni-rich NiTi shape memory alloy. Mater. Manuf. Process..

[27] Chaudhari R., Vora J., Parikh D.M. (2020). Multi-response Optimization of WEDM Parameters Using an Integrated Approach of RSM–GRA Analysis for Pure Titanium. J. Inst. Eng. India Ser. D.

[28] Thangaraj M., Annamalai R., Moiduddin K., Alkindi M., Ramalingam S., Alghamdi O. (2020). Enhancing the Surface Quality of Micro Titanium Alloy Specimen in WEDM Process by Adopting TGRA-Based Optimization. Materials.

[29] Sheth M., Gajjar K., Jain A., Shah V., Patel H., Chaudhari R., Vora J. (2021). Multi-objective optimization of inconel 718 using Combined approach of taguchi—Grey relational analysis. Advances in Mechanical Engineering.

[30] Alam M.N., Siddiquee A.N., Khan Z.A., Khan N.Z. (2022). A comprehensive review on wire EDM performance evaluation. Proc. Inst. Mech. Eng. Part E J. Process Mech. Eng..

[31] Patel S., Fuse K., Gangvekar K., Badheka V. (2020). Multi-response optimization of dissimilar Al-Ti alloy FSW using Taguchi-Grey relational analysis. Key Engineering Materials.

[32] Chaudhari R., Vora J.J., Prabu S.M., Palani I., Patel V.K., Parikh D. (2019). Pareto optimization of WEDM process parameters for machining a NiTi shape memory alloy using a combined approach of RSM and heat transfer search algorithm. Adv. Manuf..

[33] Vora J., Patel V.K., Srinivasan S., Chaudhari R., Pimenov D.Y., Giasin K., Sharma S. (2021). Optimization of Activated Tungsten Inert Gas Welding Process Parameters Using Heat Transfer Search Algorithm: With Experimental Validation Using Case Studies. Metals.

[34] Chaudhari R., Khanna S., Vora J., Patel V.K., Paneliya S., Pimenov D.Y., Wojciechowski S. (2021). Experimental investigations and optimization of MWCNTs-mixed WEDM process parameters of nitinol shape memory alloy. J. Mater. Res. Technol..

[35] Nain S.S., Garg D., Kumar S. (2018). Investigation for obtaining the optimal solution for improving the performance of WEDM of super alloy Udimet-L605 using particle swarm optimization. Eng. Sci. Technol. Int. J..

[36] Sharma N., Khanna R., Gupta R.D. (2015). WEDM process variables investigation for HSLA by response surface methodology and genetic algorithm. Eng. Sci. Technol. Int. J..

[37] Phate M.R., Toney S.B. (2019). Modeling and prediction of WEDM performance parameters for Al/SiCp MMC using dimensional analysis and artificial neural network. Eng. Sci. Technol. Int. J..

[38] Deb K., Member A., Pratap A., Agarwal S., Meyarivan T. (2002). A Fast and Elitist Multiobjective Genetic Algorithm: NSGA-II. IEEE Trans. Evol. Comput..

[39] Khullar V.R., Sharma N., Kishore S., Sharma R. (2017). RSM- and NSGA-II-Based Multiple Performance Characteristics Optimization of EDM Parameters for AISI 5160. Arab. J. Sci. Eng..

[40] Kumar K., Agarwal S. (2012). Multi-objective parametric optimization on machining with wire electric discharge machining. Int. J. Adv. Manuf. Technol..

[41] Krishnan S.A., Samuel G.L. (2012). Multi-objective optimization of material removal rate and surface roughness in wire electrical discharge turning. Int. J. Adv. Manuf. Technol..

[42] Golshan A., Gohari S., Ayob A. Modeling and optimization of cylindrical wire electro discharge machining of AISI D3 tool steel using non-dominated sorting genetic algorithm. Proceedings of the 2011 International Conference on Graphic and Image Processing.

[43] Bezerra M.A., Santelli R.E., Oliveira E.P., Villar L.S., Escaleira L.A. (2008). Response surface methodology (RSM) as a tool for optimization in analytical chemistry. Talanta.

[44] Maruyama S.A., Palombini S.V., Claus T., Carbonera F., Montanher P.F., de Souza N.E., Visentainer J.V., Gomes S.T.M., Matsushita M. (2013). Application of Box-Behnken design to the study of fatty acids and antioxidant activity from enriched white bread. J. Braz. Chem. Soc..

[45] Ahmad A., Alkharfy K.M., Wani T.A., Raish M. (2015). Application of Box-Behnken design for ultrasonic-assisted extraction of polysaccharides from *Paeonia emodi*. Int. J. Biol. Macromol..

[46] Zhang G., Li W., Zhang Y., Huang Y., Zhang Z., Chen Z. (2020). Analysis and reduction of process energy consumption and thermal deformation in a micro-structure wire electrode electric discharge machining thin-wall component. J. Clean. Prod..

[47] Chen Z., Zhou H., Wu C., Zhang G., Yan H. (2022). A New Wire Electrode for Improving the Machining Characteristics of High-Volume Fraction SiCp/Al Composite in WEDM. Materials.

[48] Chen F., Peng J., Lei D., Liu J., Zhao G. (2013). Optimization of genistein solubilization by κ-carrageenan hydrogel using response surface methodology. Food Sci. Hum. Wellness.

[49] Ilavenil K.K., Pandian P., Kasthuri A. (2023). Adsorption study of removal of lead ions using *Prosopis juliflora* and prediction by artificial neural network modeling. Mater. Today Proc..

[50] Morelli L.L., Prado M.A. (2012). Extraction optimization for antioxidant phenolic compounds in red grape jam using ultrasound with a response surface methodology. Ultrason. Sonochem..

